# Early detection of external cervical resorption in posterior teeth: a radiographic, cross-sectional study of an adolescent population

**DOI:** 10.1259/dmfr.20220223

**Published:** 2023-01-03

**Authors:** Julie Suhr Villefrance, Ann Wenzel, Lise-Lotte Kirkevang, Michael Væth, Jennifer Christensen, Louise Hauge Matzen

**Affiliations:** 1 Section for Oral Radiology, Department of Dentistry and Oral Health, Aarhus University, Aarhus, Denmark; 2 Department of Public Health, Aarhus University, Aarhus, Denmark; 3 Danish Community Dentistry for children and adolescents, Aalborg, Denmark

**Keywords:** Bitewings, external cervical resorption, diagnosis, prevalence

## Abstract

**Objectives:**

To establish the prevalence and severity of external cervical resorption (ECR) in posterior teeth observed in bitewing (BW) radiographs in an epidemiological study of a 17-year-old patient population from community dentistry. Furthermore, to assess the potential predisposing factors for ECR.

**Methods:**

Posterior BWs from 5596 patients (2717 females, 2879 males; mean age 17.8 years) were assessed by three observers in order to detect ECR (using Heithersay’s classification system, severity classes 1–4). When ECR was suspected, cone beam CT (CBCT) was offered to verify diagnosis. Prevalence was estimated based on ECR suspected in BWs and finally in CBCT. Possible predisposing factors (orthodontic treatment, trauma, and periodontal disease) were recorded and assessed for association with ECR.

**Results:**

In 41 patients, ECR was suspected in BWs (suspected prevalence 0.73%). 32 patients accepted CBCT examination, of which eight were verified to have ECR (final prevalence 0.18%). In 24 patients, other disease entities and abnormal tooth morphology, that had mimicked ECR in BWs, excluded ECR in CBCT. ECR severity ranged from class 1–3 in BW and 2–4 in CBCT. All but one case had not been diagnosed by the patient’s community dentist. No statistically significant association between predisposing factors and ECR was identified.

**Conclusions:**

ECR had low prevalence in this adolescent population, as observed in both BWs and CBCT. Still, early detection of ECR is important for treatment prognosis, and attention should be paid to this disease entity when assessing BWs obtained for other diagnostic purposes. CBCT may subsequently aid in verifying the disease.

## Introduction

External cervical resorption (ECR) appears as an invasive damage of the dental hard tissues, and in most cases, it has its entry point in the cervical area of the tooth, hence the name. The loss of hard tissue is caused by pathologic odontoclastic activity.^1^ The surface lesion(s) (portals of entry) initiated by odontoclastic activity is/are small in the beginning.^2^ Further on in the resorptive process, ECR will spread in the vertical direction both coronally and apically and can lead to additional surface lesions (portals of exit) if the resorptive cells perforate the periodontal ligament in the root.^1,3–5^ Clinically, ECR can present as an irregular surface cavity, sometimes with a pink spot observed clinically through the dental hard tissues, and often there will be bleeding on probing at the entry point of the resorptive process.^2,6^ In most cases though, neither clinical features nor symptoms reveal the presence of ECR before an incidental finding in an intraoral radiograph. The intraoral image shows irregular radiolucent striae in the dentine originating from the cervical area. In more severe cases, the striae may exceed into the root and the crown of the tooth and in severely affected cases penetrate the pericanalar resorption-resistant sheet (PRRS), thereby gaining access to the pulp.^1^ Besides the vertical extent of ECR, in more severe cases, often there is circumferential spread around the pulp as well.^7^

Several studies have assessed the potential predisposing factors of ECR. In general, orthodontic treatment, trauma to the tooth, and possibly periodontal treatment have been found to be associated with ECR.^8–10^ ECR seems therefore to be a multifactorial disease that does not exhibit a predilection for either sex.^6,11,12^ Studies have shown a wide age distribution of ECR from 18 to 81 years (mean 45.77 years),^10^ 12–89 years (mean 50.9 years),^12^ 10–65 years,^9^ and 11–75 years (mean 37 years),^8^ but for all the mentioned studies, the number of individuals in the young age groups has been small. It seems that different age groups are associated with different predisposing factors.^9,10^ For example, in older age groups, periodontal treatment appeared to be a predisposing factor,^10^ and in younger age groups, the predisposing factor seemed to be either trauma or orthodontic treatment.^9^ Also, there was a low number of Heithersay severity class 1 or 2 compared to the dominating representation of class 3 and 4 in all of these studies.^8–10,12^

Proper diagnosis and classification of ECR makes treatment planning more precise. To obtain the best prognosis, early detection of ECR is important since previous studies have shown that treatment may be successful in less severe cases, whereas treatment may not even be possible in late disease stages.^3,13,14^ Despite the growing interest for ECR, there is a lack of scientific knowledge in the form of longitudinal clinical and epidemiological studies on ECR.^5,15–19^ In community dentistry for children and adolescents, there has been little focus on detection and treatment of ECR, and the prevalence and severity of the disease in a population of children and adolescents is unknown. It seems reasonable to assume that symptomless ECR lesions are present already in an adolescent population, diagnosis may however demand special knowledge, since many factors, anatomical structures as well as other disease entities, may mimic ECR in its early stages.^8–10,12^ In community dentistry for children, radiographic images, particularly bitewings (BW), may be available for most children, recorded with the purpose of caries diagnosis.^20^ Early diagnosis of ECR may be obtained based on these radiographs if the clinician knows what to look for. Furthermore, a list of differential diagnostic examples in radiographs might ease clinicians’ correct diagnosis. It seems that no studies have focused on this aspect.

The aim of this study was to establish the prevalence and severity of ECR in posterior teeth observed in BW radiographs in an epidemiological study of a 17-year-old patient population from community dentistry. It is hypothesized that the prevalence of ECR in posterior teeth will be low. Furthermore, to assess predisposing factors related to ECR in posterior teeth and to determine differential conditions that may mimic ECR.

## Methods and materials

### Patient population

This cross-sectional study included 5596 patients (2717 females, 2879 males) with a mean age of 17.8 years (ranging from 16.0 to 18.6). The patients were part of the Danish Community Dentistry for children and adolescents in Aalborg, Denmark, where patients are offered a final examination before leaving the community dental healthcare system at 18 years of age to enter the private dental healthcare sector.

### Information from patients’ records

Anamnestic and clinical information for each patient was obtained from the patient’s files by a clinician from the community dentistry, including: general disease (yes/no); medication (yes/no); smoking (yes/no); periodontitis (yes/no; *i.e*. at least one index tooth (first molar/lateral/central incisor) with pocket depth >4 mm); DMFS index (decayed, missing, filled surfaces); tooth/root resorption (yes/no); orthodontic treatment (yes/no); dentoalveolar surgery such as tooth removal and denudation (yes/no); tooth agenesis (yes/no); dental trauma (yes/no). If a positive score was given in the dental categories additional information of the involved tooth/teeth was/were also registered.

### Radiographic examination and assessment

When indicated during the clinical examination, a posterior BW (imaging premolars and molars, except third molars) had been taken in each side of the mouth using a storage phosphor plate system (Digora 2.8 PSP System, Soredex, Finland) with a receptor holder. The inclusion criterion was that patients had BWs in their files recorded at the final examination before dismissal from Community Dentistry for children and adolescents.

During 2017–2020, radiographic assessment was performed at the community dental clinic to resemble the normal clinical situation and to ensure that the children’s identity was not compromised. The study did not require special approval from an ethical committee, since the BW radiographs were part of an already existing radiographic guideline and part of the patient’s record in the community healthcare clinic, thus no intervention in addition to regularly planned treatment was performed. Any legal considerations were described and handled by a lawyer at the Community Dentistry. In Denmark, where this study was performed, the study has been registered and approved as part of Aarhus University united research at the Danish Data Supervision Entity. When ECR was suspected in BWs, the disease was sought verified by CBCT, which is the standard for this disease to plan the treatment.^21^ General data protection regulations were followed, which involve that no patient-specific data were extracted from the patient’s records, and all registrations were conducted at the community dentistry clinics. The patients were assigned a number for anonymization for further data treatment.

**Figure 1.**
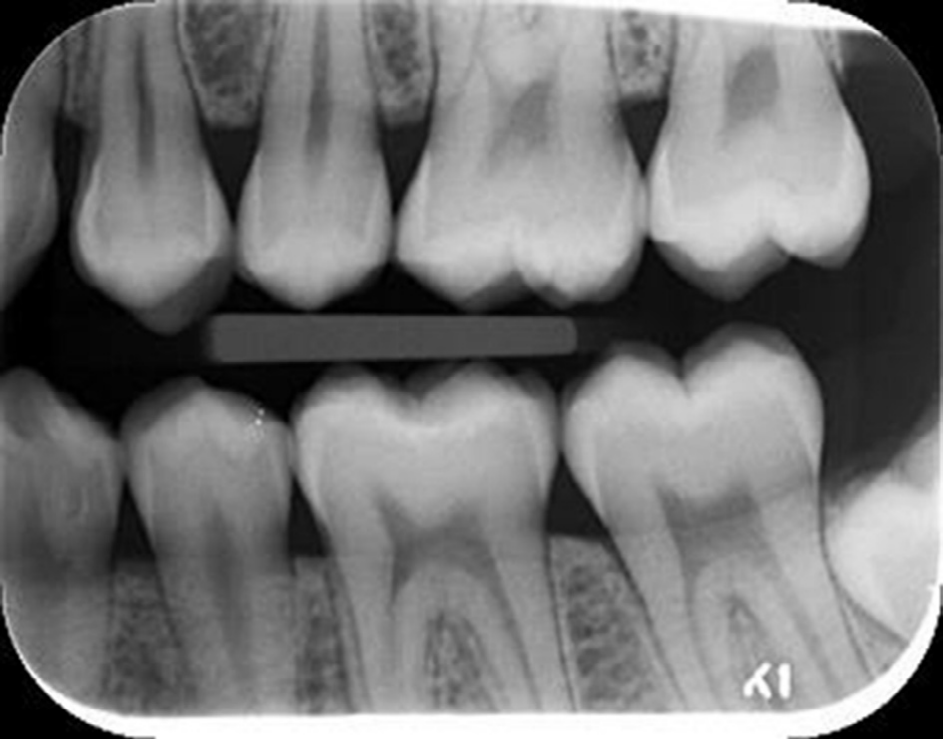
Example of a BW with eight included teeth (first and second upper premolars and molars, first and second lower premolars and molars). Tooth 34 has an enamel defect, which is too coronally positioned to be mistaken for ECR. BW, bitewing; ECR, external cervical resorption.

The radiographs were viewed on high quality monitors (Dell P2417H, China) in a clinic room with dimmed lighting using Digora or Visiquick (4.3.0.874 Citodent Imaging, B.V., Amstel 312, NL-1017 AP Amsterdam, The Netherlands) software. Various enhancement filters in Digora and Visiquick were available. The patient’s birthdate and sex were registered. If present in the image, posterior teeth from the first premolar to the second molar were registered in each side together with the date of the images. Only permanent teeth were registered. A tooth was defined as present if more than half of the tooth crown and pulp in the horizontal plane and the entire crown and cervical area in the vertical plane were visible in the image (Figure 1).

Two observers (differentiating among three dentists: two dentists with specialist radiology experience and one dentist in radiology training) assessed the BWs from the patients in consensus. Each tooth was assessed with the focus to detect ECR. If ECR was suspected, an observer with specialist endodontic background was also consulted in order to obtain consensus opinion. The observers were properly calibrated before assessing the images; *i.e*. all observers were part of a previous study^22^ regarding ECR severity assessment in radiographs of 245 teeth with ECR. The initial calibration in this study consisted of discussion of 25 cases from the previous study.

ECR severity was defined by criteria suggested by Heithersay.^8^ The Heithersay classification implies an increasing disease severity from class 1 to class 4. Class 1 is a small, well-defined resorption in the outer part of the tooth, in most cases in the cervical area. Class 2 denotes a larger defect extending further into the crown and also closer to the pulp with initiating resorption channels into the radicular dentine. Class 3 is a more dominating resorption extending into the coronal third of the root. Class 4 describes an extensive, invasive resorption present in most parts of the tooth also with the possibility of penetrating the pulp tissue. Any severity defined the tooth as suspicious of ECR.

When ECR was suspected in the BW, the patient was offered a CBCT examination (Cranex3D, Soredex, Finland; 5 × 5 cm field of view (FOV), 0.085 mm voxel) for a more detailed investigation, since a previous study has shown that CBCT provides a full circumferential view of the tooth and most often leads to a more severe ECR diagnosis than 2D images.^22^

### Data treatment and statistical analysis

Data was registered in Excel (Microsoft Office 2010, Microsoft Corp., Redmond, WA) and imported to Stata 15.1 (StataCorp, College Station, TX; release 15) for statistical analysis. Patient age was calculated by subtracting date of birth from date for BW examination.

Suspected prevalence of ECR was estimated by: suspected prevalence in BWs = (number of patients suspected with ECR/total number of patients). Final prevalence of ECR was estimated by the formula: final prevalence in CBCT = (number of patients suspected with ECR/total number of patients)*(number of patients confirmed with ECR/number of patients suspected with ECR). Cross-tables with χ^2^ test were created to check for association between ECR and the registrations from the patient’s record, especially focusing on the possible predisposing factors orthodontic treatment, trauma and periodontal disease.

## Results

**Figure 2.**
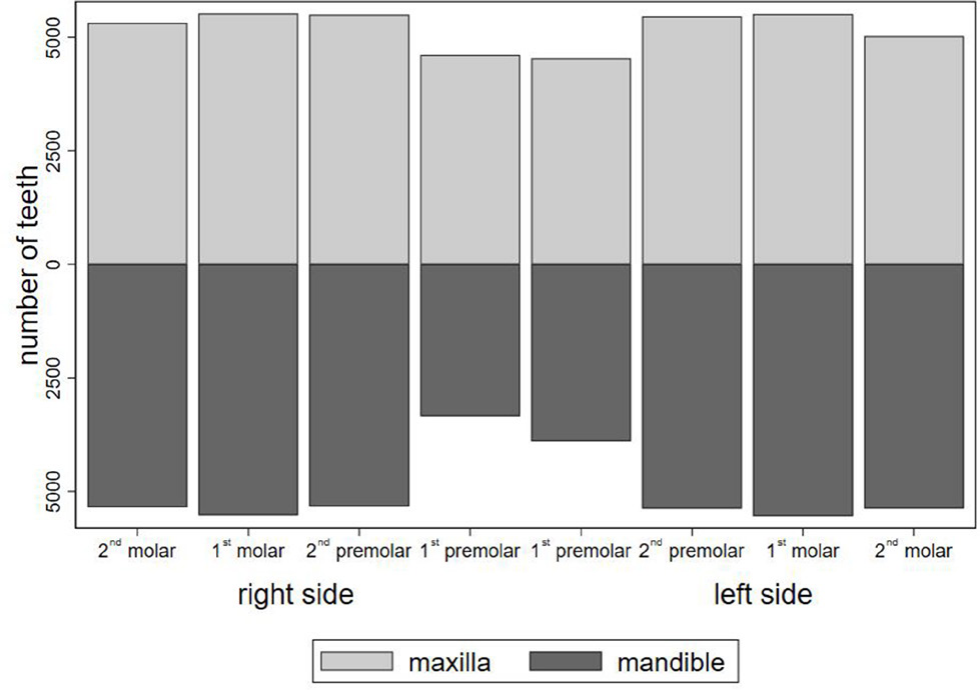
Distribution of recorded tooth types.

A total of 81,248 teeth were recorded in 5596 included patients. As seen in Table 1, all 16 teeth displayed in 2 BWs were recorded for approximately one-third of the patients; for the majority, one or more teeth could not be recorded. Figure 2 shows the distribution of the recorded tooth types. It is seen that mostly the first premolars in the mandible could not be registered in the BWs (3943 first premolars were not registered in the mandible out of 11,192 possible).

**Table 1.** Distribution of number of teeth registered in a patient and the total number of teeth

Number of teeth registered in a patient	Number of patients	Number of teeth
2	2	4
3	1	3
5	2	10
6	8	48
7	11	77
8	20	160
9	21	189
10	49	490
11	107	1177
12	383	4596
13	530	6890
14	1139	15,946
15	1510	22,650
16	1813	29,008
Total	5596	81,248

Of the 5596 patients, ECR was suspected in a BW image in 41 patients (45 teeth). Four patients had two teeth with ECR suspicion while the rest of the suspected patients had one tooth. The prevalence of patients suspected to have ECR in posterior teeth based on a BW was therefore 0.73%. All but one of the suspected ECRs had not been diagnosed previously by the patients’ dentists. In Table 2, the Heithersay classification established in consensus by three observers of the 45 teeth suspected with ECR can be seen. The majority of teeth were assessed with Heithersay class 1.

**Table 2.** Consensus Heithersay classification of the 45 teeth suspected with ECR in BWs

Heithersay score in BW	1	2	3	Total
Number of teeth with suspected ECR	20	13	12	45

BW, bitewing; ECR, external cervical resorption.

**Figure 3.**
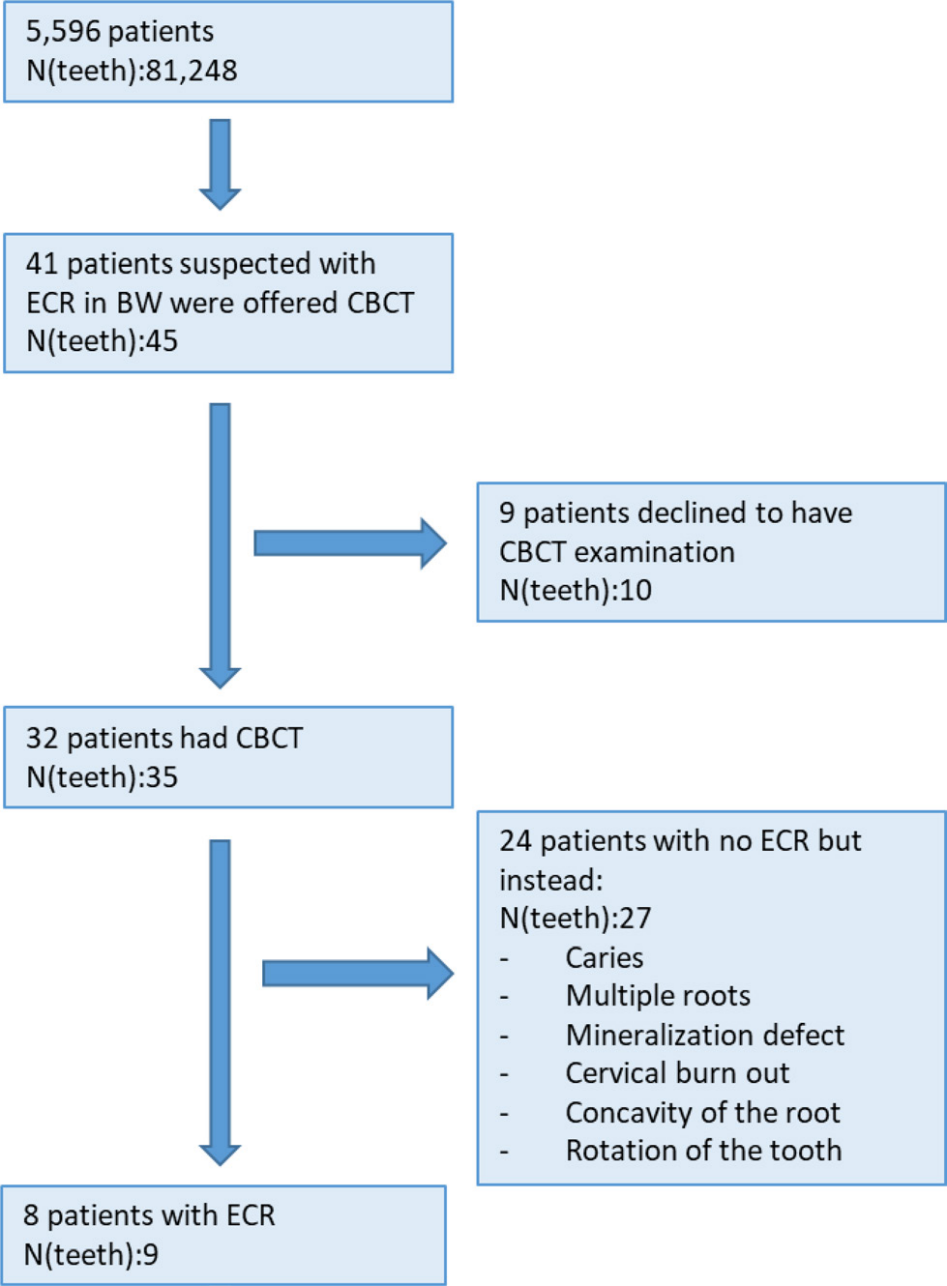
Flowchart of the study.

**Figure 4.**
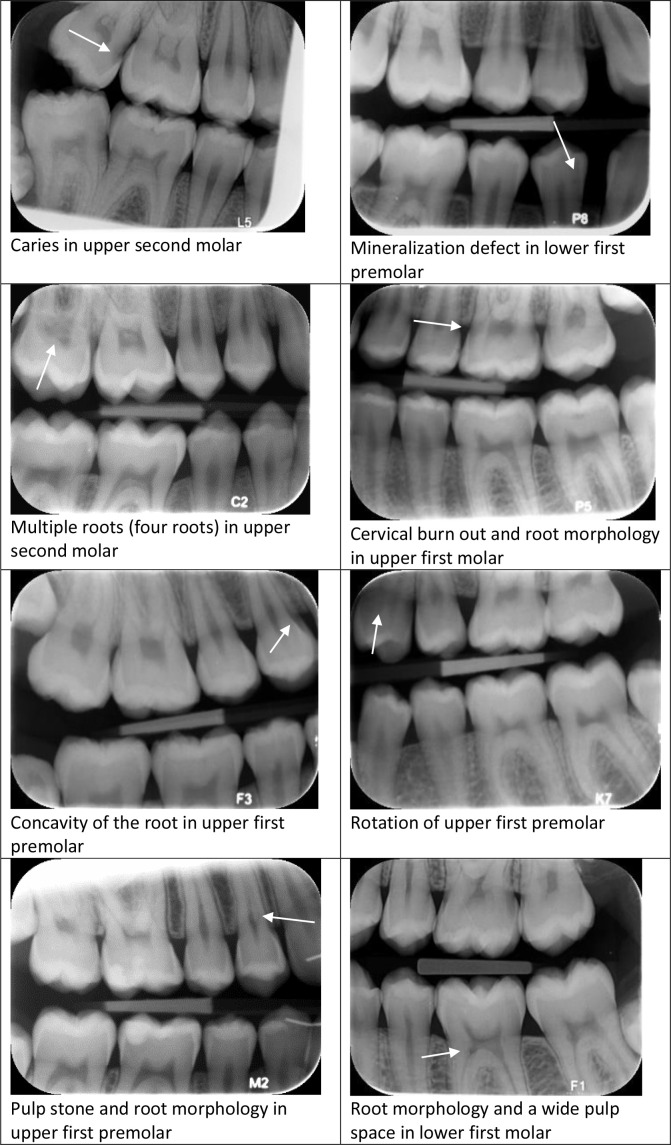
Panel of various structures and diseases that were identified as differential diagnosis to ECR, leading to a rejection of the ECR diagnosis. ECR, external cervical resorption.

A CBCT was offered to all patients with suspected ECR in BWs. Nine patients (ten teeth) declined a CBCT examination while 32 patients (35 teeth) accepted a CBCT examination. Eight patients (nine teeth) were diagnosed with ECR after CBCT, including one extra tooth diagnosed with ECR in the CBCT sections, which was not detected in the patient’s BWs, defined as a false-negative diagnosis in BWs. In 24 patients (27 teeth), the suspected ECR diagnosis was rejected (false-positive diagnoses) because CBCT revealed differential conditions that mimicked ECR, such as a caries lesion, multiple roots with varying anatomy, mineralization defects, cervical burn out, concavity of the root, rotation of the tooth etc. (flowchart Figure 3 and Figure 4). Based on the CBCT examination, the final prevalence of patients with ECR (*n* = 8) in the present population was 0.18% (95%CI: 0.09%, 0.36%).

**Figure 5.**
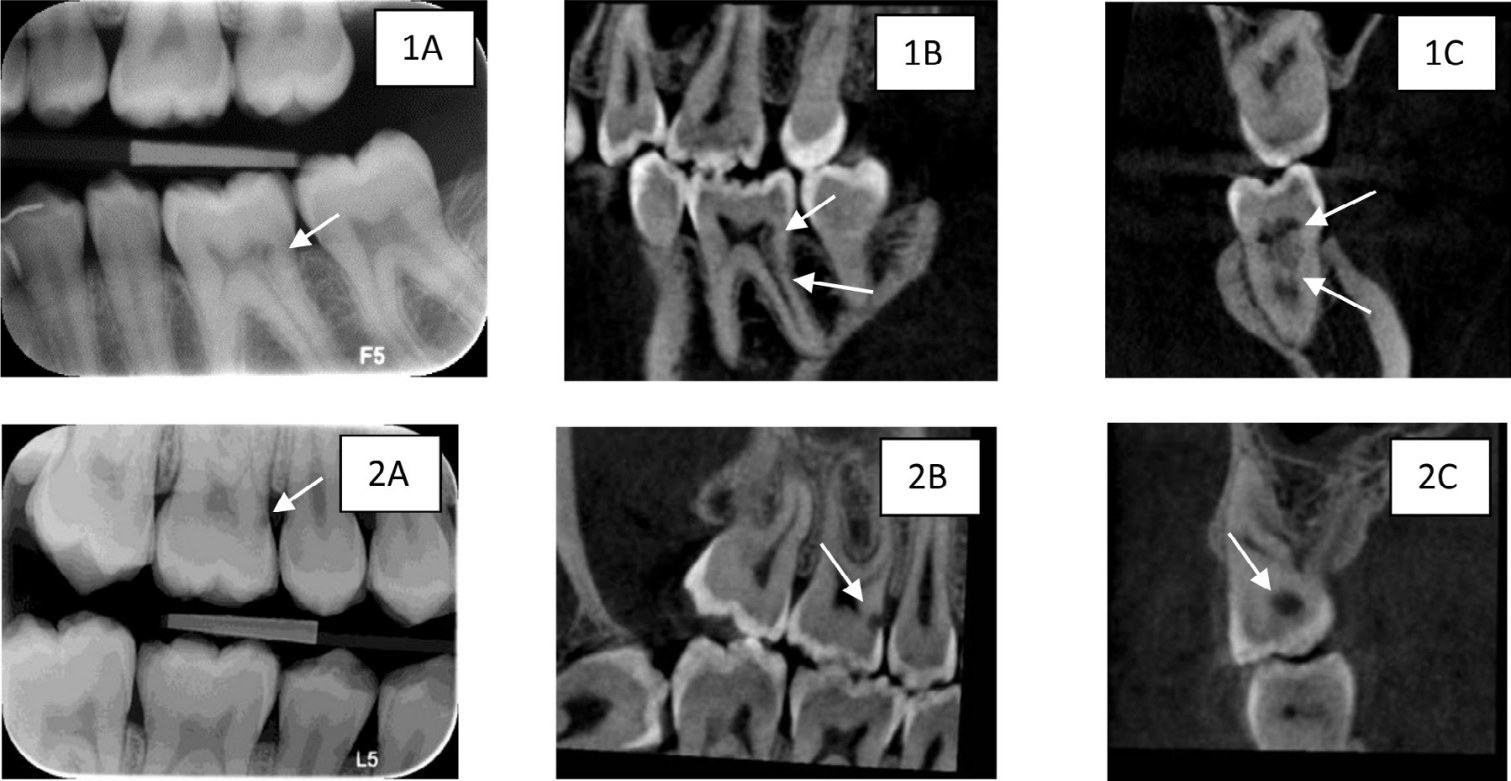
Example of ECR that changes severity assessment from BW to CBCT. Case 1: a lower left side molar where severity assessment changed from class 3 in BW to class 4 in CBCT. 1A: BW; 1B: CBCT section in the sagittal plane; 1C: CBCT section in the coronal plane. Case 2: an upper right side molar where severity assessment changed from class 1 in BW to class 2 in CBCT. 2A: BW; 2B: CBCT section in the sagittal plane; 2C: CBCT section in the coronal plane. BW, bitewing; CBCT, cone beam CT; ECR, external cervical resorption.

**Figure 6.**
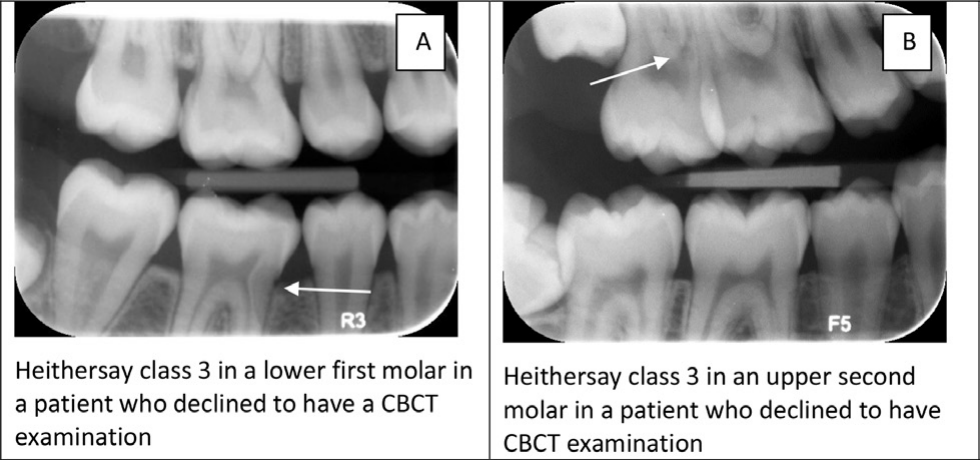
Examples of teeth suspected with ECR where the patient declined a CBCT examination. (A) ECR is suspected in the lower first molar (arrow); (B) ECR is suspected in the upper second molar (arrow). CBCT, cone beam CT; ECR, external cervical resorption.

The severity of ECR diagnosed in CBCT was scored in consensus by the three observers. Comparing the severity of ECR in BWs and CBCT images, five teeth changed to a more severe category in CBCT, while four obtained the same severity score. See Table 3 for severity of ECR and change from BW to CBCT for eight patients with nine teeth diagnosed with ECR in both BW and CBCT. The table also shows the distribution of severity class for those teeth where CBCT examination rejected the ECR diagnosis (false positive diagnosis); most of these were Heithersay class 1 in BW. Figure 5 shows an example of change of severity class from BW class 3 to CBCT class 4 in an lower first molar and a change of severity class 1 in BW to class 2 in CBCT in an upper first molar.
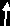
The distribution of teeth in patients who declined the CBCT examination was five teeth with class 1, three teeth with class 2, and two teeth with class 3 severity in BW. Examples of patients who declined to have a CBCT are seen in Figure 6. Both teeth were scored as a class 3 in BWs.

**Table 3.** Consensus of Heithersay classification in BW and CBCT for the patients who accepted a CBCT examination

Heithersay classification	BW class 0	BW class 1 (13 rejected)	BW class 2 (8 rejected)	BW class 3 (6 rejected)	Total (27 rejected)
CBCT class 2	1	2	2	0	5
CBCT class 3	0	0	0	2	2
CBCT class 4	0	0	0	2	2
Total	1	2	2	4	9

BT, bitewing; CBCT, cone beam CT; ECR, external cervical resorption.

Numbers in brackets are those teeth where the ECR diagnosis was rejected after CBCT examination.

Trauma was registered in 1189 patients, mostly in frontal teeth. 10 patients both had trauma and ECR registered in one tooth, but in different areas in the mouth (traume to the frontal teeth, *e.g*. upper right insicor and resorption in upper left premolar). Orthodontic treatment was registered in 1438 patients at different levels (both removable and fixed appliances), but no statistically significant association between orthodontic treatment and ECR was identified. In 149 patients, periodontitis was registered. None of these patients had ECR, and therefore no association was identified. No statistically significant associations were identified between any of the anamnestic or clinical registrations and ECR.

## Discussion

The population in this cross-sectional, retrospective epidemiological study was considered to reflect the young adult Danish population. Both rural and city areas were represented. Patient data were registered over a period of almost 4 years. The study considered a rare but relevant disease, external cervical resorption. It is noteworthy that this disease entity in some textbooks of Dentomaxillofacial Radiology is treated in a rather summary manner. The terminology is inconsistent, and some textbooks do not distinguish between, *e.g*. apical resorption and ECR; both go under the diagnosis *external resorption* ^23^ while others use a more explanatory terminology, which includes the origin of the lesion.^24^ Likewise in previous papers,^3,25^ ECR is referred to by various names: odontoclastoma, idiopathic external resorption, invasive cervical resorption, subepithelial inflammatory root resorption, etc. In more recent literature, authors seem however to have reached consensus on the nomenclature, external cervical resorption,^11,14^ a term that we also encourage and advocate.

Prevalence of a disease is defined as the proportion of individuals in a population with that specific disease.^26^ In the present study, the suspected prevalence of ECR based on BWs of posterior teeth (premolars and molars) was 0.73%. Several factors may contribute to an underestimation (false-negative recording) of the suspected prevalence. First, it was not possible to include all 16 teeth in every patient, thereby more patients might have had suspicion of ECR if all possible teeth in the patient were recordable. If every patient had 16 teeth recorded in the BWs, an additional 8288 teeth would have been included in the study. Assuming that the observed frequencies of ECR in teeth applies, this would lead to 4–5 additionally suspected ECR, of which one would be verified by CBCT. Second, the applied formula has as an implicit assumption that no teeth with ECR truly exist among the unsuspected teeth, *i.e*. that the classification of the teeth in BWs resulted in no false-negative decisions. However, an extra tooth with ECR was in fact found in the CBCT volume of a suspected tooth by the observers in the study, so false-negative decisions in BWs cannot be ruled out. To quantify the size of the underestimation from this, omission would require CBCT examinations of a sample of patients with no suspicion of ECR, but an additional radiation exposure of patients with no suspicion of ECR would not be justified, thus this is not possible. Third, some patients declined to undergo a CBCT examination of a suspected tooth, thereby presumably also underestimating the final prevalence (0.18%). Since this study was on a disease with low prevalence, a high number of false-positive diagnosis may theoretically also be expected (false-positive fraction is highly dependent on disease prevalence). The number of false-positive recordings (ECR suspected in BW but rejected by CBCT) was higher though in the beginning of the study than in the end, which means that there was a steep learning curve, even for the experienced observers in this study. There are many differential diagnostic conditions that the dentist needs to recognize in radiographs (Figure 4), and more awareness and knowledge of the special appearance of ECR is therefore needed. The radiographic findings should also not stand alone, but a clinical examination should be included.^14^ Conditions like mineralization defects of the crown and morphological concavities in the cervical area represented by a radiolucency in a radiograph can often be clinically visible, and thereby part of the CBCT examinations may be avoided.

Despite these drawbacks, our study is novel since it is the first with a truly epidemiological design. A few previous studies have published an estimate of ECR prevalence.^8,12,27^ These estimates were, however, not obtained with proper epidemiological designs. One study described the prevalence of invasive cervical resorption (now termed external cervical resorption) to be 0.02%.^8^ The author calculated a prevalence on the basis of 257 teeth in 222 patients referred for treatment of ECR in Adelaide, Australia, a city of 1.2 million citizens. Apparently, the authors’ calculations were based on the formula prevalence = (persons with ECR/citizens in the area). With no systematic collection of data, prevalence might be underestimated due to missing cases with ECR. It seems that the author only assessed patients referred to him, and thereby no information on untreated cases and cases treated by general dentists or other specialists in the area was obtained. If all patients came from Adelaide, then the denominator may be relatively accurate, but the numerator could be problematic/incomplete. Another study included a total of 11 patients with ECR over a period of 8 years and stated a low prevalence of 0.08%.^27^ There was no description of the source population, which thereby makes it difficult to find out how the prevalence was calculated, or whether it could be the incidence rate instead. Incidence usually expresses the fraction of new cases with a disease within a time period.^26^ In a third study, a combined prevalence of 2.3% was described.^12^ These authors found a total of 98 teeth in 76 patients with ECR at a university graduate endodontics clinic over a 10-year period, but no description of the underlying population was given.

In the present study, it should be born in mind that the estimated prevalence is valid for posterior teeth only and not for the entire dentition. In one-third of the patients, all 16 possible teeth were recorded in the BWs. In two-thirds of the patients, one or more teeth were not sufficiently imaged; when missing, it was mostly first premolars in the lower jaw. Other studies have described the difficulties in obtaining a perfect posterior BW in every recording.^28^ All BWs in the present study were recorded with the purpose of caries diagnostics. In community dentistry, no anterior BW is however recorded if no (or only small) caries lesions are observed in the posterior BW, even when the first premolar is missing in the image. A study has shown that when no lesions are observed in molars and second premolars, there is an extremely low risk that lesions are present in first premolars.^28^ Additionally, some patients also had agenesis of premolars and some had one or more teeth removed because of caries or in association with orthodontic treatment. In the literature, ECR seems often to occur in the upper frontal teeth,^9,22^ which may be associated with the high trauma frequency in these teeth.^29^ In this study, more than one-fifth of the included patients had had a trauma to the upper frontal teeth, which is suggested to be a predisposing factor for ECR, however this not likely to have an impact on posterior teeth in the individual. Thus, no statistically significant associations between any of the recorded predisposing factors (trauma, orthodontics and marginal periodontitis) and ECR were identified in the present study. However, due to the small number of verified ECR, the study lacked statistical power to detect an association documented in previous studies.^8–10^

In recent years, CBCT has become an efficient radiographic method for the clinician to establish a proper diagnosis of specific oral diseases.^30^ CBCT has been shown effective in viewing ECR in a three-dimensional perspective, sometimes revealing more circumferential spread than observed in 2D images and also several surface entries.^22^ There are limitations of CBCT to bear in mind though. CBCT may still underestimate the true extent of ECR because it is not possible to see the interface between the different stages of resorption and repair.^1,2^ To observe the true extent of ECR, a combination of Nano computed tomography (Nano CT) and hard tissue histological images may be the gold- standard.^1,2^ This, of course, is not possible in the clinical situation, where CBCT seems to be a beneficial diagnostic method though, adding to the clinical and 2D radiographic examination. The additional information from CBCT may influence the severity classification of the disease and thereby the treatment decision.^22,31,32^ A former study has shown that ECR generally was scored as more severe in CBCT than in 2D periapical images using the Heithersay classification.^22^ This was also the case in the present study, where five teeth changed to a more severe class from BW to CBCT, while four remained in the same severity class. In a BW examination, only the crown, cervical area, and the coronal part of the roots are imaged. Consequently, a class 4 ECR may not be possible to identify in a BW since this is defined as a lesion that penetrates into more than the coronal third of the root complex. Some of the class 3 scores in BWs may therefore have been class 4 scores, if they had been observed in a periapical 2D image.

A pioneer study on ECR by Heithersay in 2004 showed that tooth survival rate was related to severity: the higher the ECR class, the lower the survival rate.^3^ A recent study by Mavridou et al^14^ showed that a clinical approach strategy also considering pain sensation, probing feasibility, and the presence of bone like structure along with classification seemed to be of importance for treatment and prognosis of the tooth. From both studies, it seems that the prognosis is highly dependent on early detection of the lesion. It is therefore believed that for those patients with a true-positive diagnosis of ECR (ECR verified in CBCT), the diagnosis made a difference since these patients can be treated early with a high survival prognosis for the tooth.

## Conclusions

ECR has a low prevalence in posterior teeth in this adolescent population as observed in both BWs (suspected prevalence of ECR was 0.73%) and CBCT (final prevalence of ECR was 0.18%). Still, early detection of ECR is important for treatment prognosis, and attention should be paid to this disease entity when assessing BWs obtained for other diagnostic purposes. CBCT may subsequently aid in verifying the disease. ECR was assessed to be more severe in CBCT compared to BWs.
